# Impaired Heat Shock Response in Cells Expressing Full-Length Polyglutamine-Expanded Huntingtin

**DOI:** 10.1371/journal.pone.0037929

**Published:** 2012-05-23

**Authors:** Sidhartha M. Chafekar, Martin L. Duennwald

**Affiliations:** Regenerative Biology Program, Boston Biomedical Research Institute, Watertown, Massachusetts, United States of America; University of Florida, United States of America

## Abstract

The molecular mechanisms by which polyglutamine (polyQ)-expanded huntingtin (Htt) causes neurodegeneration in Huntington's disease (HD) remain unclear. The malfunction of cellular proteostasis has been suggested as central in HD pathogenesis and also as a target of therapeutic interventions for the treatment of HD. We present results that offer a previously unexplored perspective regarding impaired proteostasis in HD. We find that, under non-stress conditions, the proteostatic capacity of cells expressing full length polyQ-expanded Htt is adequate. Yet, under stress conditions, the presence of polyQ-expanded Htt impairs the heat shock response, a key component of cellular proteostasis. This impaired heat shock response results in a reduced capacity to withstand the damage caused by cellular stress. We demonstrate that in cells expressing polyQ-expanded Htt the levels of heat shock transcription factor 1 (HSF1) are reduced, and, as a consequence, these cells have an impaired a heat shock response. Also, we found reduced HSF1 and HSP70 levels in the striata of HD knock-in mice when compared to wild-type mice. Our results suggests that full length, non-aggregated polyQ-expanded Htt blocks the effective induction of the heat shock response under stress conditions and may thus trigger the accumulation of cellular damage during the course of HD pathogenesis.

## Introduction

Abnormally expanded polyglutamine (polyQ) regions within nine different proteins cause nine different neurodegenerative diseases, including the Spinocerebellar Ataxias and Huntington's disease (HD) [1], [2]. In HD, a polyQ expansion in the protein huntingtin (Htt) leads to progressive neurodegeneration resulting in detrimental symptoms, such as impaired movement, cognition, and behavioral function [3], [4]. On a pathological level, polyQ-expanded Htt accumulates in ubiquitinated inclusions in the cytosol and nucleus, predominantly in neurons of the striatum and cortex of HD patients [5]. The striatum and cortex are also most affected by neurodegeneration in HD. Even though the genetic basis of HD is clear, the cellular mechanisms by which polyQ-expanded Htt causes the dysfunction and the demise of neurons remains perplexing. The toxicity associated with polyQ-expanded Htt has been attributed to the disturbance of numerous cellular pathways, including impaired vesicular transport, ER stress, impaired transcription, and impaired proteostasis [6], [7], [8], [9], [10], [11], [12].

Cellular mechanisms of proteostasis, i.e. all cellular processes that regulate the accurate production, maintenance, and degradation of proteins, antagonize many toxic effects associated with polyQ-expanded Htt [13]. Molecular chaperones and heat shock proteins are major components of cellular proteostasis. The heat shock response, an evolutionary conserved cellular response to diverse kinds of cellular stresses, is central to the induction of molecular chaperones and other heat shock proteins. HSF1 (heat shock transcription factor 1) is a major transcriptional regulator of the heat shock response in eukaryotes [14].

Compounds that elicit the heat shock response have been suggested to have therapeutic benefits in neurodegenerative diseases, including HD [15], [16], [17]. For example, the small molecules geldanamycin and celastrol can confer protection from polyQ toxicity by activating the heat shock response [18], [19]. Both celastrol and geldanamycin increase the activity of the transcription factor HSF1 [19], [20]. Further, Neef et al. identified a small molecule that effectively activated HSF1, induced a solid heat shock response, and reduced polyQ toxicity [16]. In a high-through-put screen, Calamini at al. identified small molecules that elicit a heat shock response. Many of these small molecules also reduced polyQ toxicity [21]. Likewise, using HD mouse models, Labbadia et al. showed that treatment with an Hsp90 inhibitor can protect from polyQ toxicity by the partial activation of a heat shock response. They also provide evidence that the brains of mice expressing polyQ-expanded Htt have a reduced capacity to mount a heat shock response upon treatment with the Hsp90 inhibitor compared to wild-type mice [22]. These results imply that activating HSF1 is a promising therapeutic strategy for the treatment of HD.

We investigated the role of heat shock response and the role of HSF1 in cellular and mouse models of HD. For our studies, we chose murine striatal neuron-derived cells that express full length polyQ-expanded Htt at physiological levels (STHdh(Q111)) and the corresponding wild-type cells (STHdh(Q7)) [23]. In these cells, polyQ-expanded Htt does not produce any detectable insoluble protein aggregates and, under normal growth conditions, these cells do not show any detectable levels of polyQ toxicity [23], [24], [25]. These features are in stark contrast to HD models that overexpress amino-terminal fragments of the polyQ-expanded Htt proteins (e.g. exonI fragments) that are associated with prominent polyQ aggregation and, in many cases, with strong polyQ toxicity. The intricate role of polyQ aggregation in HD pathogenesis has been the center of a substantial number of studies und remains puzzling [26], [27], [28]. Because of the absence of these drastic features of polyQ misfolding, the STHdh(Q111) cells may serve as cellular models for specific and early events in HD pathogenesis, which may precede polyQ fragmentation, polyQ aggregation, and polyQ toxicity [29].

We used STHdh(Q111) cells to ask how full-length polyQ-expanded Htt modulates the heat shock response. We found that the presence of full-length polyQ-expanded Htt sensitizes cells to heat shock and other stressors. Further, our results demonstrate that cells expressing full-length polyQ-expanded Htt inhibited the induction of central heat shock proteins and accumulated increased amounts of damaged proteins when exposed to stress. Also, full–length polyQ-expanded Htt expressing cells had reduced levels of HSF1. The inability to mount a functional heat shock response thus uncovers polyQ toxicity under conditions of cellular stress.

Our study provides novel insights into how full-length polyQ-expanded Htt impairs the heat shock response and cellular proteostasis, and thus sensitizes cells to stress. It is plausible that this sensitization may be a critical contributor to early HD pathogenesis possibly even before the occurrence of polyQ aggregation.

## Results

### Full length polyQ-expanded Htt sensitizes cells to a heat shock

**Figure 1:**
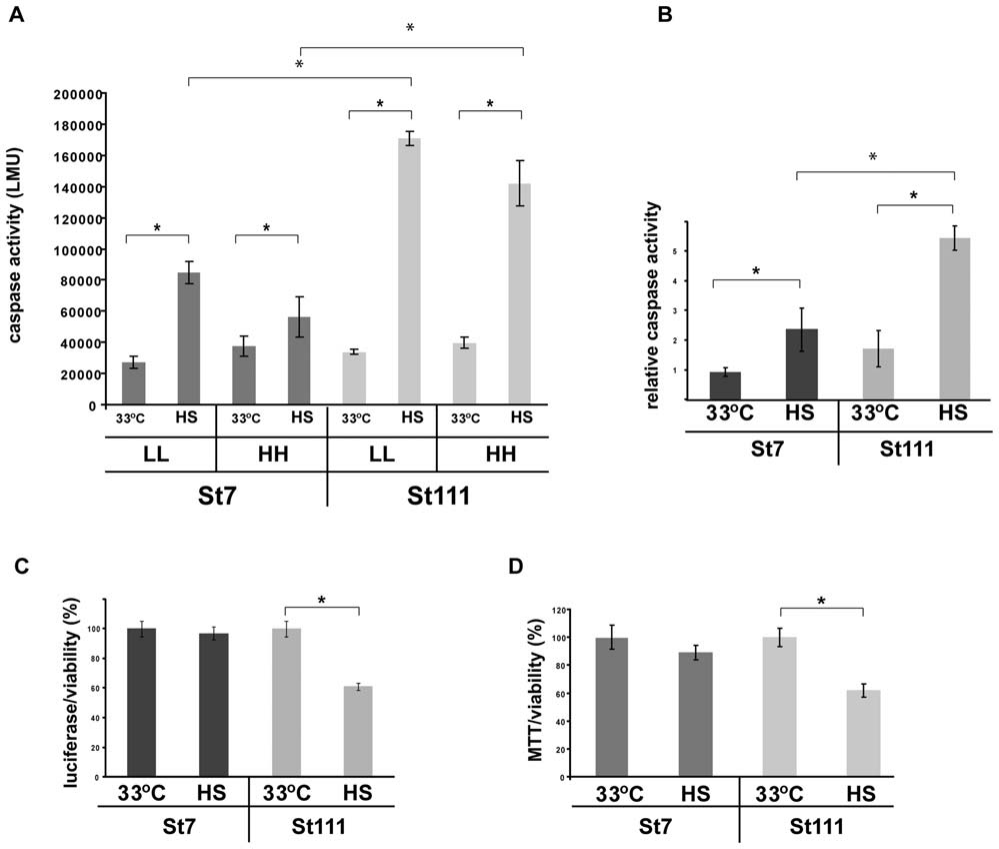
Decreased resistance to a heat shock in cells expressing polyQ-expanded Htt. **A**) Caspase assay of cells that were grown at 33°C and of heat shocked cells (six hours at 42°C). In either medium containing high concentrations of glucose and serum (HH) or lower concentration of glucose and serum (LL). The error bars represent SDs. Results from four independent experiments were analyzed. * p<0.1 (two tailed t-test). **B**) Relative caspase activities (from Figure 1 A). The values for non heat shocked cells were set as 1 and the other signals were calculated accordingly. **C**) Viability assay (luciferase activity, Promega) of STHdh(Q7) and STHdh(Q111) cells that were grown at 33°C and of heat shocked cells (six hours at 42°C). The signals from STHdh(Q7) cells grown at 33°C was set as 100% and the other signals were calculated accordingly. The error bars represent SDs. Results from four independent experiments were analyzed. * p<0.05 (two tailed t-test). **D** MTT assay of STHdh(Q7) and STHdh(Q111) cells that were grown at 33°C and of heat shocked cells (six hours at 42°C). The signals from STHdh(Q7) cells grown at 33°C was set as 100% and the other signals were calculated accordingly. The error bars represent SDs. Results from four independent experiments were analyzed. * p<0.05 (two tailed t-test). Experiments shown under B), C), and D) were carried out using LL medium.

We first tested whether the expression of full-length polyQ-expanded Htt in the striatal cell line (STHdh(Q111)) sensitized cells to a heat shock. To this end, we monitored caspase activity in STHdh(Q7) (wild-type) and STHdh(Q111) cells under normal growth conditions (i.e. growth at 33°C) and upon heat shock (i.e. growth at 42°C for six hours, Figure 1). Caspase activity, which indicates the induction of apoptosis [30], served as a proxy for reduced cellular viability.

We monitored the impact of a heat shock on viability of STHdh(Q7) and STHdh(Q111) cells in two different media (Figure 1 A). We used media that contained a high level of glucose and serum (HH, 4.5 g/l and 10% respectively) and we used media that contained a lower level of glucose and serum (LL, 1 g/l and 1% respectively). In LL media the cells did not divide anymore unlike cells in the HH medium (data not shown). The LL medium thus allows us to avoid complications that may arise due to the presence of a temperature sensitive T-antigen that was used to create the STHdh(Q7) and STHdh(Q111) cell lines. These different growth media did not cause any differences in cellular viability when the cells were incubated for 24 hours at 33°C (Figure 1 A and B).

We measured increased caspase activity in STHdh(Q111) cells upon heat shock compared to STHdh(Q7) cells in the HH medium (Figure 1 A). Notably, this sensitivity to a heat shock is not altered by the LL medium, indicating that these growth conditions do not significantly modulate sensitivity to heat shock conditions. We therefore used the LL medium for all ensuing experiments throughout this study.

We measured an approximately twofold increase in caspase activity upon heat shock in the STHdh(Q7) cells compared non-heat shocked STHdh(Q7) cells. The increase was substantially higher (approximately eightfold) in heat shocked STHdh(Q111) cells compared to non-heat shocked STHdh(Q111) cells (Figure 1 A and B). To confirm our results with the caspase assay, we measured the viability of cells by a luciferase/ATP assay which uses cellular ATP levels as a proxy for cellular viability (Figure 1 C) [23]. In this assay, relative ATP levels dropped by approximately 40% in STHdh(Q111) cells upon heat shock, whereas in STHdh(Q7) cells ATP levels remained unaltered by a heat shock. Another well-established method to determine cellular viability is the measurement of respiratory activity by an MTT (3-(4,5-dimethylthiazol-2-yl)-2,5-diphenyltetrazolium bromide) assay [31]. We also detected an approximate 40% decrease in respiratory activity in heat shocked STHdh(Q111) cells (Figure 1 D). STHdh(Q7) cells were not significantly affected. Based on these three independent assays for cellular viability we conclude that expression of full-length polyQ-expanded Htt sensitized cells to a heat shock.

### Full length polyQ-expanded Htt impairs the heat shock response

We next asked whether cells expressing polyQ-expanded Htt have a reduced ability to elicit an efficient heat shock response. To test this hypothesis, we monitored the levels of major heat shock proteins (Hsps) in cells expressing polyQ-expanded Htt and compared those to wild-type cells at regular growth temperature (33°C) and upon a short heat shock (42°C for three hours). Notably, the three hour heat shock heat shock does not produce the strong reduction of cellular viability as observed for the six hour heat shock (data not shown). Consequently, the milder heat shock conditions allowed us to monitor the heat shock response in cells that do not yet display overt signs of cellular stress and reduced viability.

**Figure 2:**
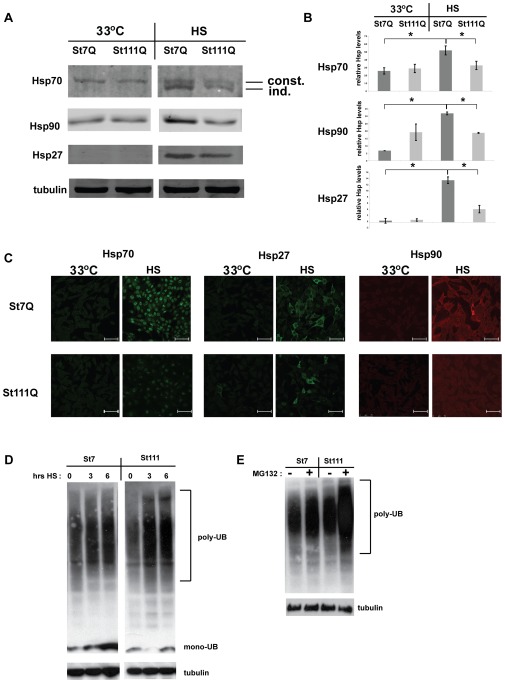
Impaired heat shock response in cells expressing polyQ-expanded Htt. **A**) Western blots were prepared with protein lysates from STHdh(Q7) (St7Q) and STHdh(Q111) (St111Q) cells that were grown at 33°C or heat shocked (HS), i.e. grown at 33°C and then incubated at 42°C for three hours. The blots were probed with the indicated anti-Hsp antibodies and anti-tubulin antibodies as a loading control. The anti-Hsp70 antibody (3A3) detects both constitutive (const.) and inducible (ind.) Hsp70s. **B**) Quantification of Western blots as shown in Figure 1 A. All signals were normalized to the corresponding tubulin signal in each experiment. For Hsp70, both the constitutive and the inducible Hsp70s were quantified together. The error bars present SDs. * p<0.01 (two-tailed t-test). **C**) Immunofluorescence microscopy of fixed cells that were grown at 33°C and heat shocked cells (HS, three hours at 42°C) using the indicated anti-Hsp antibodies. The scale bars represent 75 µm **D**) Western blot probed with an anti-ubiquitin antibody of protein lysates derived from STHdh(Q7) and STHdh(Q111) cells that were grown at 33°C or exposed to a heat shock (42°C) for either three or six hours. A western blot using an anti-tubulin antibody (bottom) served as a loading control. E) Western blot probed with an anti-ubiquitin antibody of protein lysates derived from STHdh(Q7) and STHdh(Q111) cells that were grown at 33°C in the absence or presence of 10 µM MG132. A western blot using an anti-tubulin antibody (bottom) served as a loading control.

We monitored the levels of major Hsps (Hsp70s, Hsp90, and Hsp27) in STHdh(Q7) cells and in STHdh(Q111) cells by Western blotting and immunofluorescence microscopy. The anti-Hsp70 antibody (3A3) that we used for these experiments detects both the inducible and the constitutive variants of Hsp70 (Figure 2 A) [32], [33]. We observed that the levels of these Hsps and several other Hsps in both STHdh(Q7) and in STHdh(Q111) were very low in comparison to many other cell lines. In particular, cell lines derived from tumors typically have much higher Hsp levels (N2a, PC12, SH-SY5Y, not shown).

Under regular growth conditions (33°C) the Hsp levels in STHdh(Q7) and STHdh(Q111) were equally low. When subjected to heat shock (HS, 42°C for three hours), STHdh(Q7) cells showed the expected increase in Hsp levels. By contrast, heat shock had a much smaller effect on Hsp levels in STHdh(Q111) cells (Figure 2 A). The quantification of these results revealed that while in STHdh(Q7) cells there was an approximately three-fold increase in Hsp70 levels (both Hsp70 versions combined), six-fold increase in Hsp90 levels, and 18-fold increase in Hsp27 levels, there was little to no increase in the levels of any of these Hsps in STHdh(Q111) cells (Figure 2 B). This impaired induction of the expression of Hsp70s, Hsp27, and Hsp90 was confirmed by immunofluorescent microscopy (Figure 2 C). Collectively, these results demonstrated a severe defect in the induction of major Hsps in cells expressing full-length polyQ-expanded Htt.

We hypothesized that the inability to mount a heat shock response in STHdh(Q111) would elicit the accumulation of heat damaged proteins. Such damaged proteins often accumulate as ubiquitinated protein species. We monitored ubiquitinated proteins in STHdh(Q7) and STHdh(Q111) cells that were either grown at 33°C or exposed to heat shock for extended periods of time at 42°C. Western blot analysis (Figure 2 D) shows that both STHdh(Q7) and STHdh(Q111) cells accumulate a “smear” of higher molecular weight, ubiquitin-positive proteins upon heat shock. STHdh(Q111) accumulated a higher amount of poly-ubiquitinated proteins than STHdh(Q7) cells indicating impaired processing of damaged proteins in these cells upon heat shock. We also observed a stronger accumulation of ubiquitin positive proteins after treatment with the proteasome inhibitor MG132 in STHdh(Q111) cells than in STHdh(Q7) cells (Figure 2 E).

**Figure 3:**
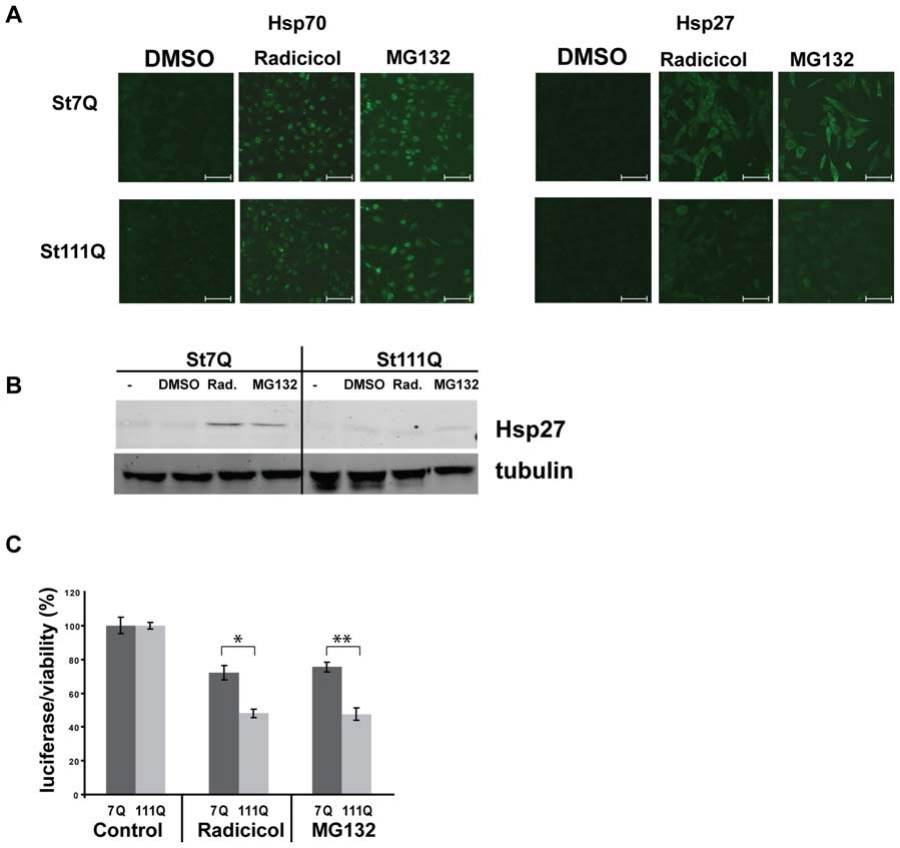
Decreased resistance to inhibition of the proteasome or Hsp90 in cells expressing polyQ-expanded Htt. **A**) Immunofluorescence microscopy of STHdh(Q7) and STHdh(Q111) cells that were treated for six hours with the Hsp90 inhibitor radicicol (5 µM) or the proteasome inhibitor MG132 (10 µM) using anti-Hsp70 and anti-Hsp27 antibodies. The scale bars represent 75 µm **B**) Western blots prepared from protein lysates treated as in A. **C**) Viability assay (luciferase activity, Promega) of STHdh(Q7) and STHdh(Q111) cells that were treated with DMSO (control), radicicol (5 µM), or MG132 (10 µM) for six hours. The error bars represent SD. Results from four independent experiments were analyzed. * p<0.001 and **p<0.005 (two tailed t-test).

In addition to heat shock, we used the proteasome inhibitor MG132 and the Hsp90 inhibitor radicicol to induce a heat shock response (Figure 2 E and Figure 3 A and B). Previous studies have established that MG132 and radicicol elicit a robust heat shock response in mammalian cells [23], [34], [35]. Our immunofluorescence microscopy revealed that treatment with MG132 or radicicol increased Hsp70s and Hsp27 levels in STHdh(Q7) cells (Figure 3 A). By contrast, in STHdh(Q111) cells treated with MG132 or radicicol, Hsp70s and Hsp27 show only slightly elevated levels of Hsp70s and Hsp27 (Figure 3 A). Western blot analysis showed the same lack of Hsp induction in STHdh(Q111) cells treated with MG132 and radicicol. Particularly, the induction of Hsp27 appeared to be impaired in STHdh(Q111) cells (Figure 3 B).

We also examined the viability of STHdh(Q7) and STHdh(Q111) cells after treatment with MG132 or radicicol (Figure 3 C). We noticed that STHdh(Q7) cells were highly sensitive to both drugs even at low concentrations. Nevertheless, STHdh(Q111) cells were significantly more sensitive than STHdh(Q7) cells, decreasing ATP levels in STHdh(Q111) by approximately 20% compared to STHdh(Q7) cells. These results demonstrated that the polyQ-expanded Htt cells are highly sensitized to MG132 and radicicol treatment. Note that the increased sensitivity of STHdh(Q111) cells to MG132 corresponded to a stronger accumulation of ubiquitinated proteins in these cells compared to STHdh(Q7) cells. The specific sensitivity of STHdh(Q111) to inhibition of Hsp90 by radicicol appeared to disagree with previous results in different HD models [19], [22]. Importantly, in contrast to this previous studies, we were using an HD cell model expressing full length polyQ-expanded Htt not a model overexpressing polyQ-expanded Htt fragments.

**Figure 4:**
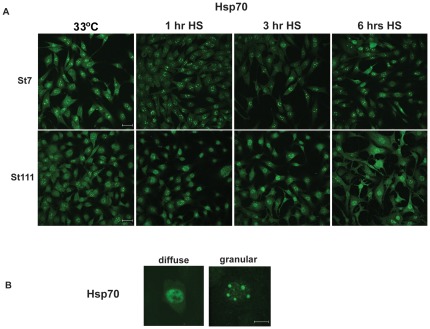
Aberrant Hsp70 localization upon heat shock in cells expressing polyQ-expanded Htt. **A**) Immunofluorescence microscopy of fixed cells that were grown at 33°C and heat shocked cells (HS, 42°C) for either one, three, or six hours probed with anti-Hsp70 antibodies. The scale bars represent 75 µm. The 33°C samples were exposed longer to visualize Hsp70 localization. **B**) Immunofluorescence microscopy of fixed cells as in Figure 4 A. Pictures were taken at a higher magnification (scale bars represent 15 µm) to demonstrate the difference between diffuse and granular Hsp70 staining in the nucleus.

We next monitored the localization of Hsp70s in STHdh(Q7) and STHdh(Q111) cells that were exposed to heat shock for a short period of time (one hour) or longer periods of time (three and six hours, Figure 4 A). After one hour and particularly after three and six hours at 42°C, STHdh(Q7) cells showed Hsp70 localized in nuclear granules whereas a higher proportion of STHdh(Q111) cells showed more diffuse Hsp70 staining in the nucleus and the cytosol. This nuclear granular Hsp70 staining (Figure 4 B) may be reminiscent of nuclear stress granules [36] and may indicate a particular nuclear stress response that is functional in STHdh(Q7) cells yet is defective in STHdh(Q111) cells. To allow the visualization of the lower levels of Hsp70, the pictures of non-heat shocked cells (33°C, left panels of Figure 4 A) were taken with higher exposure times (double) than the ones shown in Figure 2 B and Figure 3 A.

The experiments in Figure 2, 3 and 4 imply that striatum-derived cells have generally low levels of certain Hsps. These cells may thus replicate the previously described low levels of molecular chaperones and Hsps in certain neurons in HD mouse models and their sensitivity to protein folding stress [37], [38]. These low levels of Hsps are adequate to maintain proper proteostasis in non-stressed cells. Yet, under stress, cells that express full-length polyQ-expanded Htt (STHdh(Q111)) have an impaired heat-shock response, accumulate a higher amount of damaged proteins, and are therefore highly sensitized to proteostatic stress, such as heat shock, proteasome or Hsp90 inhibition. Thus, cellular stress uncovers a deleterious effect on proteostasis of full-length polyQ-expanded Htt.

### Reduced HSF1 levels in cells expressing polyQ-expanded Htt

We speculated that the impaired heat shock response in STHdh(Q111) cells may be caused by impaired activation of HSF1. HSF1 is the major heat shock transcription factor and controls the expression of many Hsps, mainly in response to stress [14], [39]. In mammalian cells, HSF1 is activated through multiple interdependent steps, including phosphorylation, trimerization, and dissociation from molecular chaperones (Hsp70 and Hsp90), sumoylation, and translocation to the nucleus [14]. We aimed to determine whether any of these HSF1 activation steps were impaired in cells expressing full-length polyQ-expanded Htt.

**Figure 5:**
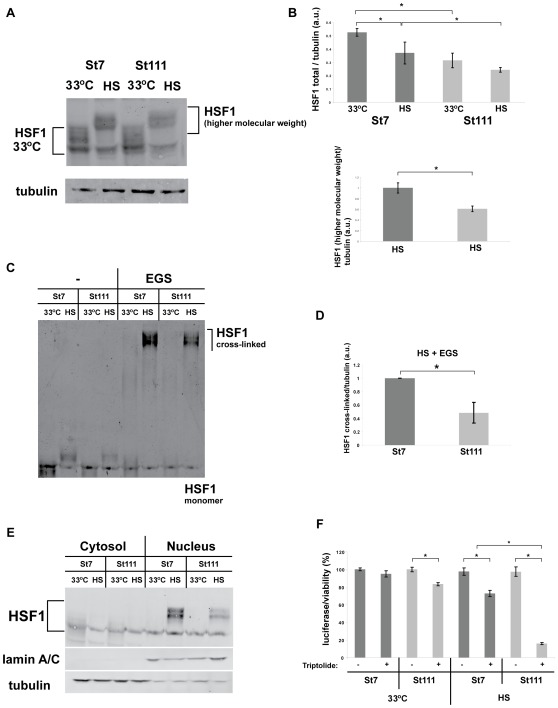
Reduced HSF1 levels in cells expressing polyQ-expanded Htt. **A**) Western blots prepared with protein lysates from STHdh(Q7) and STHdh(Q111) cells that were grown at 33°C or heat shocked (HS, 42°C for three hours). The blots were probed with anti-HSF1 antibodies and anti-tubulin antibodies as a loading control. **B**) Upper panel: quantification of Western blots as shown in Figure 5 A. Signals of total HSF1 were normalized to the corresponding tubulin signal in each experiment. Lower panel: quantification of Western blots as shown in Figure 5 A. Signals of the top, i.e. the heat shock-induced part of HSF1 were normalized to the corresponding tubulin signal in each experiment. The signal of the top part of HSF1 at 33°C was set as 1 for each condition. Error bars represent SDs. Results from three independent experiments were analyzed. *p<0.07 (two-tailed t-test). **C**) Cross-linking of HSF1. Protein lysates were prepared as in A) followed by cross-linking with EGS (see Materials and Methods for details). The ensuing Western blots were probed with and anti-HSF1 antibody. **D**) Quantification of three independent experiments as shown in C). The error bars represent SDs. * p<0.01 (two-tailed t-test). **E**) Protein lysates were prepared as in A) and then separated into cytosolic and nuclear fractions. The blots were probed with anti-HSF1 antibodies and anti-lamin A/C antibodies and tubulin antibody as a loading control and as a control for the purity of the fractions (nucleus and cytosol respectively). **F**) Viability assay (luciferase activity, Promega) of STHdh(Q7) and STHdh(Q111) cells that were grown at 33°C and heat shocked cells (three hours at 42°C) in the absence or presence of triptolide. The signal from STHdh(Q7) cells grown at 33°C without triptolide was set as 100% and the other signals were calculated accordingly. The error bars represent SDs. Results from four independent experiments were analyzed. * p<0.001 (two-tailed t-test).

Our Western-blot analysis showed the typical increase in higher molecular weight species upon heat shock in STHdh(Q7) cells and STHdh(Q111) cells (Figure 5 A). These higher molecular weight HSF1 signals conceivably represent different post-translationally modified (mostly phosphorylated) versions of heat-activated HSF1 [14]. Perplexingly, we did not observe any overt differences in these higher molecular weight HSF1 species between STHdh(Q7) and STHdh(Q111) cells. Yet careful quantification of these experiments showed that STHdh(Q111) cells have lower total (all different modified and unmodified versions combined) levels of HSF1, particularly under HS conditions (Figure 5 B, upper panel). Also, the higher molecular weight, heat shock-induced HSF1 species were significantly reduced (by approximately 40%) in heat shocked STHdh(Q111) cells compared to STHdh(Q7) cells (Figure 5 B, lower panel).

Cross-linking experiments also showed nearly identical higher HSF1 molecular weight species (possibly cross-linked HSF1 trimers and Hsps cross-linked to HSF1 [14]) in STHdh(Q7) and STHdh(Q111) cells (Figure 5 C). The amount of higher HSF1 molecular weight species was significantly reduced (by approximately 40%) in heat-shocked STHdh(Q111) cells in comparison to STHdh(Q7) cells (Figure 5 D). Also, fractionation experiments and their quantification did not detect any gross defect in nuclear translocation of HSF1 upon heat shock in STHdh(Q111) cells. (Figure 5 E). Importantly, in the experiments demonstrating both nuclear translocation and trimerization (or complex formation) of HSF1 (Figure 5 C–E), HSF1 levels were significantly lower in STHdh(Q111) cells than in STHdh(Q7) cells, particularly following a heat shock.

Our results do not conclusively rule out the possibility that certain HSF1 modifications are specifically altered or subtly modulated in STHdh(Q111) cells compared to STHdh(Q7) cells upon heat shock. Yet our results imply that none of the multiple steps involved in HSF1 activation is severely impaired in STHdh(Q111) cells, a conclusion which recapitulates the findings of Labbadia et al. [22]. The most notable difference between STHdh(Q7) and STHdh(Q111) cells in our experiments, was the lower overall level of HSF1 in STHdh(Q111) cells, particularly under heat shock conditions (Figure 5 A–E).

We also monitored the effect of triptolide on STHdh(Q7) and STHdh(Q111) cells under normal growth conditions and after heat shock (Figure 5 F). Triptolide has been reported to inhibit the heat shock response in mammalian cells at a transcriptional level [40]. We treated STHdh(Q7) and STHdh(Q111) cells with triptolide and measured cellular viability using ATP luciferase assays (Figure 5 F). At 33°C, triptolide reduced the luciferase signal in STHdh(Q111) by about 20% when compared to STHdh(Q7) cells. Upon heat shock, triptolide-treated STHdh(Q7) cells showed only slightly reduced luciferase activity. The combination of heat shock and triptolide-treatment showed a severe reduction of luciferase activity, indicating a strongly increased sensitivity of triptolide-treated STHdh(Q111) cells to heat shock. Please note that the heat shock in these experiments was shorter (only three hours at 42°C) than the heat shock in Figure 1 (six hours at 42°C) in order to detect the synthetic toxic effects of heat shock combined with triptolide treatment.

### Reduced HSF1 and Hsp70 levels in HD knock-in mice

We next explored whether HSF1 levels are reduced in HD knock-in mouse models, which represent a faithful model of HD [41]. This HD mouse model shows a slower progression of disease-related phenotypes than observed in mice engineered to overexpress polyQ-expanded Htt exonI fragments, and may thus provide insights into the pre-manifest disease state prior to massive polyQ aggregation. Of note, in this HD mouse model, the first obvious HD-related phenotypes occur after approximately twelve months of age [41].

We compared HSF1 levels in protein lysates derived from striata and cerebella of either wild-type mice or HD knock-in mice at twelve months of age. In HD and in mouse models of HD, the striatum is one of the most severely affected regions of the brain whereas the cerebellum remains mostly unaffected [42]. We therefore expected most obvious changes in HSF1 levels in the striatum, whereas the cerebellum should be less affected or not affected at all.

**Figure 6:**
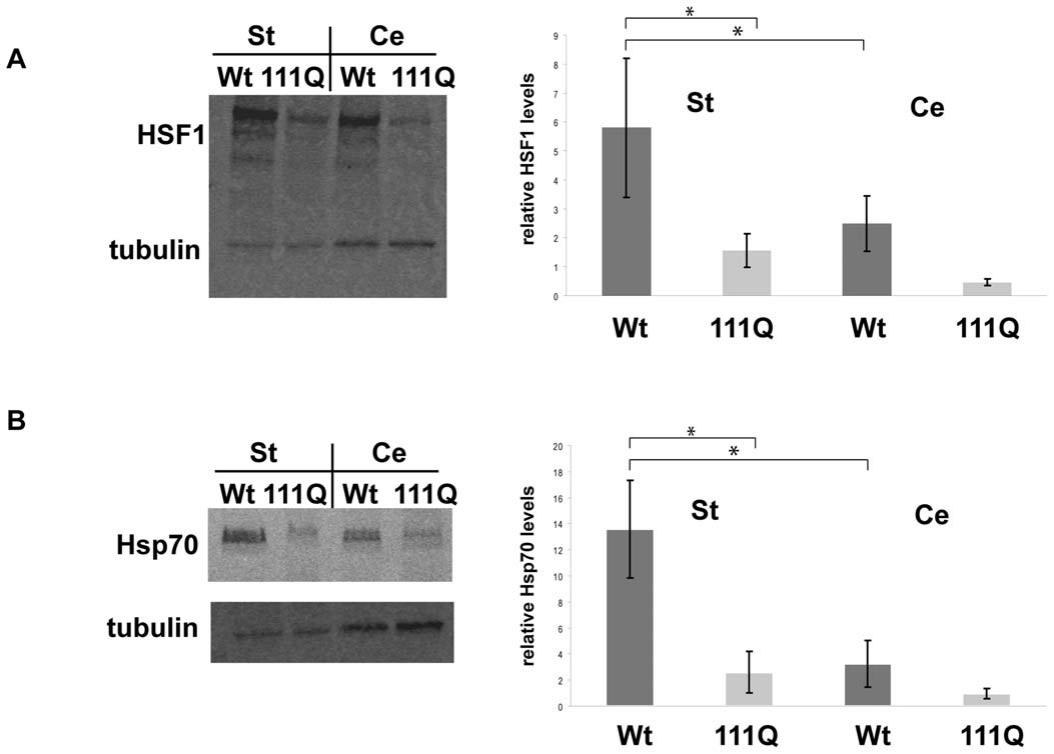
Reduced levels of HSF1 and Hsp70s in striata of HD knock-in mice. **A**) Western blots were prepared with protein lysates from the cerebella (Cer) or striata (St) from wild-type (WT) or STHdh(Q111) knock-in mice and probed with anti-HSF1 antibodies and anti-tubulin antibodies as a loading control (left panel). Quantification of three independent Western blots. Error bars represent SDs. *p<0.07 (two-tailed t-test). **B**) Western blots were prepared with protein lysates from the cerebella (Cer) or striata (St) from wild-type (WT) or STHdh(Q111) knock-in mice and probed with anti-Hsp70 antibodies (3A3) and anti-tubulin antibodies as a loading control. Quantification of three independent Western blots. Error bars represent SDs. *p<0.07 (two-tailed t-test).

Unexpectedly, Western-blot analyses showed that HSF1 levels are reduced in both the striatum and in the cerebellum of HD knock-in mice compared to wild-type mice (Figure 6 A). In the striatum and in the cerebellum, HSF1 levels are reduced by approximately 80% in HD knock-in mice compared to wild-type mice. Importantly, however, quantification of these Western-blots documented that the absolute reduction of HSF1 levels is much more severe in the striatum than in the cerebellum because in wild-type mice, HSF1 levels are much higher in the striatum than the cerebellum. Therefore, our results indicated a specific reduction of HSF1 levels in the striata of HD knock-in mice in comparison to wild-type mice.

We also explored the levels of Hsp70 in the striatum and in the cerebellum of twelve-month-old wild-type and HD knock-in mice (Figure 6 B). When comparing wild-type to knock-in mice, Hsp70 levels in the striatum were reduced by approximately 80% and in the cerebellum by about 70% in the knock-in mice. Yet, in parallel to our observations regarding HSF1, the levels of Hsp70s were more severely affected in striata because Hsp70 levels were much higher in the striatum than in the cerebellum of wild-type mice but Hsp70 levels were comparable in HD knock-in mice in striata and in cerebella. Similar to our findings regarding HSF1 levels, this result indicated a specific reduction of Hsp70 levels in the striata of HD knock-in mice. To remain consistent with our experiments using the striatum-derived cell lines (Figure 2 A and B, Figure 3 A, and Figure 4), we used the 3A3 antibody for our experiments using protein lysates derived from mice (Figure 6) B. Therefore, we are not able to determine which specific Hsp70 showed reduced expression in HD knock-in mice. Our quantification was based upon all Hsp70 signals on the Western blots. Considering the specific Hsp70 expression patterns in the mouse brain [43] it will be interesting to specify which specific Hsp70s are reduced in HD knock-in mice.

We also compared HSF1 and Hsp70 levels in the striatum and the cerebellum of six-month-old HD knock-in and wild-type mice (data not shown). We did not detect any significant changes at this age, implying that the reduction in HSF1 and Hsp70 levels occur concomitant to early phenotypic changes in HD knock-in mice.

The results using striata and cerebella from twelve-months-old HD knock-in mice confirm our findings from STHdh(Q111) cells that striatal cells expressing polyQ-expanded Htt showed lower HSF1 levels than wild-type striatal cells. Importantly, our experiments using mouse brain tissues were performed without any additional externally applied stress other than the physiological stress occurring in specific brain regions during the course of aging [10], [11], [12]. Further, we observed reduced levels of Hsp70s specifically in the striata of HD knock-in mice indicative of a muted HSF1 induction of Hsp70 expression. These results suggest that the presence of full length polyQ-expanded Htt impairs the heat shock response in the striatum in HD knock-in mice.

## Discussion

We examined how full-length polyQ-expanded Htt modulates the heat shock response and the activity of HSF1, a central transcriptional regulator of the heat shock response and cellular proteostasis. Our results demonstrate that cells expressing full-length polyQ-expanded Htt have a severely impaired heat shock response and have reduced levels of HSF1 and as a consequence an increased sensitivity to proteostatic stress. Importantly, these effects of full-length polyQ-expanded Htt can only be observed under stress conditions as cells expressing full-length polyQ-expanded Htt that were grown under normal conditions do not display any disturbances in proteostasis. Our results thus demonstrate how cellular stress can uncover the toxicity associated with polyQ-expanded Htt.

While we were preparing this manuscript, Labbadia et al. published an intriguing study describing the impaired induction of the heat shock response by the Hsp90 inhibitor NVP-HSP990 in R6/2 mice and HdhQ150 knock in mice [22]. Like our study, they find a dysregulation of HSF1 to be central for the muted heat shock response in HD mice. They suggest disturbed chromatin remodeling as a cause for HSF1 dysregulation. While our results are in agreement with these results our data may offer additional insights.

In contrast to the HD model used in our experiments, the Labbadia study used samples from mice presenting rather severe symptoms of HD, i.e. mice that already presented high degrees of polyQ toxicity and neuronal dysfunction and neurodegeneration [22]. The cellular HD model that we used does that show any toxicity associated with polyQ-expanded Htt. In fact, this HD model has been suggested to represent earlier stages of HD, i.e. before the occurrence of massive polyQ aggregation, substantial polyQ toxicity, and the ensuing cell death [23], [24]. Our results show that full-length polyQ-expanded Htt does not interfere with regular housekeeping proteostasis, i.e. the upkeep of proteins in the absence of stress. It is only under stress conditions that proteostasis fails in cells expressing full-length polyQ-expanded Htt.

Our results imply that this failure of proteostasis under stress is caused by a failure to effectively activate the heat shock response. According to the Labbadia study, aberrant chromatin structures in cells with polyQ-expanded Htt may cause this impaired activation of heat shock response. Our results offer an additional, not mutual exclusive, explanation. Reduced levels of HSF1 may contribute to the failure to activate the heat shock response.

Our study raises a number of intriguing mechanistic questions. For instance, it remains unclear why HSF1 levels in cells expressing full-length polyQ-expanded Htt are reduced. Is this reduction a consequence of lower HSF1 expression levels or the consequence of an aberrantly swift turn-over of HSF1? Does polyQ-expanded Htt directly interfere with HSF1 or is this interaction the result of more complex physiological changes in cells expressing polyQ-expanded Htt? Based on the findings by Labbadia et al., it is tempting to speculate that HSF1 expression is impaired at a transcriptional level in HD because of aberrant chromatin structures. This HSF1 dysregulation seems to specifically reduce HSF1 levels in striatal neurons and thus leave them particularly vulnerable to proteostatic stress.

It will also be important to decipher the transcriptional basis for the defect in heat shock activation by HSF1. Is the binding to HSF1 transcriptional targets impaired in stressed cells expressing full length polyQ-expanded? How does the stress-induced transcriptional profile change due to the presence of full-length polyQ-expanded Htt? Which genes are particularly affected in their expression by the reduced HSF1 levels and do these transcriptional changes explain HD-specific phenotypes?

Based upon our data and in consideration of previously published results, we hypothesize that even very early in HD pathogenesis, i.e. preceding severe polyQ aggregation or toxicity, full-length polyQ-expanded Htt impairs the heat shock response. Upon exposure to stress, such as the oxidative stress that is the toll of neurophysiologic function of specific neurons in the striatum, neurons accumulate damaged proteins without a fully functional proteostasis to cope with them. During the course of aging, this perpetual accumulation of damaged proteins may lead to cellular dysfunction and elicit the pathological hallmarks of more advanced stages of HD, including Htt fragmentation, polyQ aggregation, and neuronal cells death. Pharmacologically augmenting the heat shock response, even at pre-symptomatic stages of HD, may thus present a powerful therapeutic intervention. Clearly, a deeper understanding of the role of HSF1, the heat shock response, and cellular stress and polyQ-expanded Htt in HD is required to validate these musings.

## Materials and Methods

### Chemicals

Tunicamycin, radicicol, triptolide, and MG132 were purchased from Sigma-Aldrich.

### Antibodies

The following antibodies were used in this study: anti-Hsp27 (Cell Signaling, Danvers, MA, USA), anti-Hsp90 (Stressgen, MI), anti-lamin A/C, anti-Hsp70 (3A3), anti-ubiquitin antibody (Covance, CA, USA), anti-HSF1 (Santa Cruz Biotechnology, CA), and anti-alpha-tubulin antibody (Cedarlane, CA). FITC-labeled and Cy5-labeled anti-mouse, anti-rabbit and anti-goat secondary antibodies for fluorescence microscopy were obtained from Jackson Immuno Research (West Grove, PA). Alexa 680-labeled secondary antibodies for Western blotting were obtained from Invitrogen (Carlsbad, CA).

### Cell culture

ST*STHdhQ7/HdhQ7^/Q7^* and ST*STHdhQ111/HdhQ111^/Q111^* striatal cells derived from wild type *STHdhQ7/HdhQ7^/Q7^* and *STHdhQ111/HdhQ111^/Q111^* knock-in embryonic mice [*23*] were used in this study. Cells were grown at 5% CO2 at 33°C in Dulbecco's modified Eagle's medium (DMEM containg 4.5 g/l glucose, from CellGro, Manassas, VA) with 10% fetal bovine serum (HyClone, Logan, UT), Pen/Strep/Glutamine and 400 µg/ml G418 (both from CellGro) – this is called HH medium. 24 hrs before starting experiments, we switched the cell to DMEM medium with 1 g/L glucose (CellGro) and 1% fetal bovine serum – this is called LL medium. Cells were only used up to passage 15. We used cells at 33°C (normal growth conditions). Heat shocked cells were incubated at 42°C for the indicated periods of time.

### Protein extraction from striatal cells

To prepare total cell lysates, cells were washed with PBS and harvested by scraping them off in RIPA lysis buffer (50 mM Tris-HCl (pH 7.4), 150 mM NaCl, 1% Triton X-100, 0.1% SDS) supplemented with complete protease inhibitor cocktail and phosphatase inhibitor cocktail (Roche). Subsequently the cells were shaken in a bead beater for three minutes followed by a mild clarifying spin (500×g for 1 min). The lysates were mixed with 6× SDS sample buffer (187.5 mM Tris-HCl, pH 6.8; 6% (w/v) SDS; 30% (v/v) glycerol; 150 mM DTT; 0.03% (w/v) bromphenol blue; 2% (v/v) β-mercaptoethanol) and boiled for 5 min. In parallel, aliquots of the protein lysates were used to perform BCA protein concentration assays (Pierce) to guarantee equal loading on SDS-PAGE. For the preparation of nuclear and cytoplasmic lysates a NE-PER Nuclear protein extraction kit (Pierce) was used following the manufacturers' protocol. HSF1 trimerization was assessed using the amine-specific cross-linker ethylene glycol bis-succinimidyl succinate (EGS, Pierce) according to manufacturers' protocol.

### Western blotting

For SDS-PAGE, protein concentrations of all samples within one experiment were equalized according to BCA assays (Pierce). Proteins were transferred to nitrocellulose membranes by the iBlot Dry Blotting system (Invitrogen). Blots were blocked in 5% non-fat dry milk in PBS for one hour at RT and subsequently incubated with primary antibodies for one hour at RT or overnight at 4°C. After washing the blots were incubated with fluorescently labeled (Alexa 480 or Alexa 680) secondary antibodies for one hour at RT. Blots were scanned using the Odyssey Imaging System (Li-Cor Biosciences, Lincoln, NE, USA). Quantifications were performed using the Odyssey Imaging System software. Data from the quantifications of the Western blots represent three independent experiments for each experimental condition. The error bars represents standard deviations (SD).

### ATP/luciferase assay, MTT assay and caspase assays

To assay the viability of cells, luciferase assays (CellTiter-Glo Luminescent Cell Viability Assay, Promega) and caspase activity assays (Caspase-Glo 3/7 Assay, Promega) were performed in 96-well plates using 10,000 cells per well. Data from viability assays represent at least three independent experiments carried out in triplicate for each experimental condition. The error bars represent standard deviations (SD). MTT assays were carried out as described [9].

### Immunocytochemistry

For immunostaining PBS-washed cells were fixed in 4% paraformaldehyde for 20 min at RT, washed with PBS, permeabilized in 0.1% Triton X-100 in PBS, washed again, and subsequently blocked in blocking buffer (0.1% bovine serum albumin (BSA) and 0.075% Glycine in PBS) for 15 min. Cells were then incubated with primary antibody (for 60 min) in blocking buffer, washed with blocking buffer and exposed to secondary antibody in blocking buffer (for 60 min). Cells were washed again, and incubated with DAPI. Coverslips with the cells were mounted on microscope slides using Vectashield (Vector Labs, Burlingame, CA, USA). Pictures of the cells were taken with a Leica TCS SP2 inverted confocal microscope (Leica Microsystems) using a 40× or 63× objective and were processed with Adobe Photoshop.
